# Work factors and smoking cessation in nurses' aides: a prospective cohort study

**DOI:** 10.1186/1471-2458-5-142

**Published:** 2005-12-27

**Authors:** Willy Eriksen

**Affiliations:** 1 Division of Epidemiology, Norwegian Institute of Public Health, Oslo, Norway; 2 Department of General Practice and Community Medicine, University of Oslo, Oslo, Norway

## Abstract

**Background:**

The prevalence of smoking in nursing personnel remains high. The aim of this study was to identify work factors that predict smoking cessation among nurses' aides.

**Methods:**

Of 2720 randomly selected, Norwegian nurses' aides, who were smoking at least one cigarette per day when they completed a questionnaire in 1999, 2275 (83.6 %) completed a second questionnaire 15 months later. A wide spectrum of work factors were assessed at baseline. Respondents who reported smoking 0 cigarettes per day at follow-up were considered having stopped smoking. The odds ratios and 95 % confidence intervals of stopping smoking were derived from logistic regression models.

**Results:**

Compared with working 1–9 hours per week, working 19–36 hours per week (odds ratio (OR) = 0.35; 95 % confidence interval (CI) = 0.13 – 0.91), and working more than 36 hours per week (i.e. more than full-time job) (OR = 0.27; CI = 0.09 – 0.78) were associated with reduced odds of smoking cessation, after adjustments for daily consumption of cigarettes at baseline, age, gender, marital status, and having preschool children. Adjusting also for chronic health problems gave similar results.

**Conclusion:**

There seems to be a negative association between hours of work per week and the odds of smoking cessation in nurses' aides. It is important that health institutions offer workplace-based services with documented effects on nicotine dependence, such as smoking cessation courses, so that healthcare workers who want to stop smoking, especially those with long working hours, do not have to travel to the programme or to dedicate their leisure time to it.

## Background

Tobacco smoking remains a major cause of disease and premature death in Western societies and is an increasing health problem in developing countries [1]. Despite the health-hazards, more than 1.2 billion people in the world are daily smokers [2].

In Norway, the prevalence of daily smoking in men peaked between 1950 and 1955 [3], followed by a steady decline [3,4], and in 2004, it was measured as 27 % [5]. In women, a top level was reached in the beginning of the 1970s [3], followed by a 30-year-long period during which the prevalence of daily smoking was relatively stable (about 32 %) [3,4], but after a decline during recent years, it was measured as 25 % in 2004 [5].

Behind these figures are hidden large differences between people with high and low level of education [4]. Whereas the prevalence of smoking in people with high level of education (college, university) has fallen sharply during the last three decades, and is now around 15 %, the prevalence rate in people with low level of education (junior high school) has not shown a decline at all, and is still around 40 %.

We do not know the causes of these differences, also seen in other developed countries [6]. The Innovation Diffusion Theory maintains that a new behaviour is first adopted by people with high level of education, and spreads through the rest of the society [7], a theory that has also been used to explain the tobacco epidemic in Norway [3]. Bourdieu [8] maintains that factors like cultural taste and patterns of consumption may be part of a system of social distinction, and it is possible that smoking behaviour may be one way people signalise their belonging to a certain social position [3]. Some researchers point out that people with high level of education may respond more favourably to health promotion campaigns than people with lower level of education [6,9].

It is possible that those with less education and less resources have less access to smoking cessation services. More daily stress and worries among people with low level of education may perhaps also contribute to the resistant smoking behaviour in this group.

Norwegian authorities have during recent decades taken a series of actions to discourage the use of tobacco products, including informational campaigns, increased tobacco taxation, a total ban of tobacco product marketing, restrictions on tobacco sales (minors are not allowed to buy or sell tobacco), and restrictions on where smoking is allowed (no smoking in public places; no smoking at workplaces, including restaurants and pubs) [3,10]. These public policy interventions have probably accelerated the decline in cigarette consumption [11], although they seem to have had limited effects on the smoking behaviour of people with low level of education.

Smoking by nursing personnel is of particular interest for several reasons. The exemplary roles of nurses are important, not only to patients, but also to the general public. Nursing personnel may also play an important advisory role, educating patients about hazards of smoking and giving advice about cessation [12,13], but nurses who smoke seem to be less willing to take part in such practice [13,14], and they are more likely to hold attitudes that might reduce the effects of their advice [14]. Nurses who smoke may also be less supportive of smoke-free policy at health-care facilities [15].

In Europe, the prevalence of smoking among nurses seems to be at about the same level as the rate in the total female population [9,14]. In Norway, there is little exact knowledge about the smoking behaviour of registered nurses, but the situation among nurses' aides (assistant nurses) is worrying; more than 40 % of Norwegian nurses' aides seem to be daily smokers [16], in sharp contrast to female physicians, with a prevalence rate lower than 10 % [17].

In the beginning of the 1990s, Norwegian health authorities developed and implemented plans to establish "smoke-free" hospitals, hoping that such actions also could reduce the proportion of smokers among hospital employees. In 2000, a study concluded that there was still a long way to go before Norwegian hospitals were really smoke-free [18]. On the initiative of the healthcare workers' unions in Norway, a nationwide anti-smoke campaign, "Smokefree hospital employees", was launched in 2003, but the effects of this comprehensive programme remain to be seen.

Research during recent decades has provided extensive knowledge of the factors that maintain regular smoking [19-23]. Pharmacological characteristics of nicotine [19], people's genetic constitution [20], and a series of psychological and social factors, such as emotional distress and exposure to temptations [21-23], seem to be of importance.

The relationship between working conditions and the occurrence of smoking cessation has been examined in a number of studies [24-35]. These studies suggest that smoking cessation may be more frequent in workers with high social support at work [24-27], and less frequent in workers with many working hours per week, shift work, high physical workload, low influence on their work situation, or frequent exposure to role conflicts at work [24,28-30,35]. Many of these studies were linked to special cessation programmes, though, and may have given an unrepresentative picture of the cessation process. The majority of quitters stop smoking on their own.

Only a few studies have examined the relationship between working conditions and the occurrence of smoking cessation in nursing personnel [33,34], and none of these focused on nurses aides. Making inferences from studies of other occupational groups may be difficult, as working conditions vary from one occupation to another. The few studies of nursing personnel so far suggest that nurses who work with dying patients, and those who do not experience conflicts with physicians, are more likely to quit smoking [33,34].

The aim of the present study was to identify physical, psychological, social, and organisational work factors that predict smoking cessation in nurses' aides who are daily smokers.

## Methods

### Study design

A prospective cohort study was conducted.

### Data collection and participants

Nursing personnel in Norway include two large occupational groups: registered nurses, with at least three years training after high school, and certified nurses' aides, with either one year training after junior high school or a course that is part of a high school program. In addition, a small group of unlicensed nurses' aides have no formal training and often hold temporary jobs. The number of vocationally active nurses' aides (both certified and unlicensed personnel) was estimated as approximately 55 000 in 1999 (Norwegian Union of Health – and Social Workers, personal communication). About 50 000 of these were members of the Norwegian Union of Health – and Social Workers (the Union).

During the last week of October, 1999, 12 000 nurses' aides were drawn randomly from the Union's list of members, and were mailed a questionnaire. The objective was to study working conditions, life-style, and health. After one reminder, 7478 (62.3 %) consented to participate and filled in the questionnaire. The list of members also included persons who had retired from working life because of age, disability, or other reasons, and contacts over telephone during the data collection gave the impression that many of these non-working individuals were not motivated for participating in the study. Hence, the true response rate of the vocationally active subjects was probably higher than the overall response rate.

The sample of the study that is presented here, was selected from the 7478 participants by the following inclusion criteria: i) being vocationally active and not on leave because of illness or pregnancy, and ii) smoking at least one cigarette per day. The first criterium was fulfilled by 6485 participants, among whom 2720 fulfilled also the second criterium. Of these 2720 nurses' aides, who comprised the sample of the present study, 2275 (83.6 %) completed also a second postal questionnaire 15 months later.

Characteristics of the individuals who filled in the questionnaire both at baseline and the follow-up are presented in Table 1. Middle-aged, married or cohabiting women comprised the majority of the sample. Participants who did not answer the follow-up questionnaire were more likely to be younger than 30, and they were more likely to report a stressful work situation (i.e. they reported higher work demands, more frequent exposure to role conflicts, more often exposure to bullying at work, and less fairness in the immediate superior's leadership) than those who responded also to the follow-up questionnaire, but there were no significant difference (p > 0.05) between respondents and dropouts for other work factors, daily consumption of cigarettes at baseline, gender, marital status, having preschool children, and being bothered by long-term health problems (data not shown).

**Table 1:** Background factors, by the proportion of respondents who had stopped smoking at follow-up. In Norwegian nurses' aides.

		Stopped smoking		
			
Baseline characteristics	N ^†^	n	Row %	P
Age				0.003
< 30	125	23	18.4	
30–39	476	58	12.2	
40–49	1029	88	8.6	
50–59	544	47	8.6	
> 59	55	6	10.9	
				
Gender				0.873
Female	2135	213	10.0	
Male	95	9	9.5	
				
Marital status				0.002
Married or cohabiting	1721	190	11.0	
Single	505	32	6.3	
				
Pregnant				0.616
No	2186	221	10.1	
Yes	11	0	0.0	
				
Have preschool children				0.028
No	1900	182	9.6	
Yes	291	40	13.7	
				
Daily consumption of cigarettes				0.000
1–9	1221	156	12.8	
10–19	913	59	6.5	
20 or more	96	7	7.3	
				
Total sample	2230	222	10.0	

N = Total number if respondents in each category. n = number of cases in each category. Row % = proportion of cases in each category. ^† ^The total number of individuals included in these analyses were somewhat lower than the number of individuals who responded to the questionnaire, because not all respondents answered all questions; there was missing information about smoking for 45 persons.

Research protocol was approved by the Committee for Medical Research Ethics. Informed written consent was given by the respondents.

### Measures of smoking

At baseline and follow-up, respondents were asked 'How many cigarettes do you smoke per day now?' Optional answers were: 0, 1–9, 10–19, and 20 or more. Respondents who reported at baseline that they were smoking 1–9, 10–19, or 20 or more cigarettes per day were included in the present study. Respondents who reported at follow-up that they were smoking 0 cigarettes per day were considered having stopped smoking. The outcome measure was the proportion of respondents who had stopped smoking at follow-up.

### Measures of working conditions

At baseline, a series of work factors were recorded. The practice area in which the aides were working (e.g. nursing home) was recorded, as well as the number of working hours per week (optional answers: 1–9, 10–18, 19–36, > 36), and the frequency of night shift (optional answers: 'never', 'sometimes', 'rather often', and 'very often').

Exposure to heavy physical work was measured with 3 questions exploring frequency of moving patients manually in bed, frequency of lifting or supporting patients manually between bed and chair, and frequency of lifting, carrying, or pushing heavy objects. The first two questions were translations of questions developed and found valid by British scientists [36].

Psychological, social, and organisational work factors were assessed by questions from the General Nordic Questionnaire for Psychological and Social factors at Work (QPSNordic) [37]. Responses were scored on Likert five-point frequency scales, except responses to the question about bullying, which had only two response options (yes and no) after a precise definition of the concept. Quantitative work demands were assessed by 4 questions (work piles up, have to work overtime, have to work in rapid pace, have too much to do). Positive challenges were assessed by 3 questions (work is challenging in a positive way, see the work as meaningful, job requires that you acquire new knowledge and skills). Role conflicts were measured with 3 questions (have to do things that you feel should be done differently, are given assignments without adequate resources, receive incompatible requests from two or more people). Control of work pace was measured with 3 questions (can set your own work pace, can decide when to take a break, can set your own working hours). Participation in important decisions was assessed by 3 questions (can choose which method to use for doing your work, can influence the amount of work, can influence decisions that are important for your work). Social support from immediate superior was assessed by 3 questions (gives support and help when needed, willing to listen, appreciates your achievements). Fairness of immediate superior's leadership was measured with 3 questions (distributes the work fairly and impartially, treats the workers fairly and equally, the relationship between you and your superior is a source of stress). Rewards for well-done work (money or encouragement) was measured with one question. Three aspects of the social climate were assessed (encouraging and supportive, distrustful and suspicious, relaxed and comfortable). Exposure to threats or violence was measured with one question. Commitment to the work unit was measured with three questions (to my friends I praise this work unit a great place to work; my values are very similar to the work unit's values; this work unit really inspires me to give my very best job). Mastery of work was also assessed by three questions (are you content with the quality of the work you do, with the amount of work you get done, with your ability to solve problems at work?). The factors that were measured with more than one question were expressed as indices, calculated as the sum of the item scores divided by the number of items (questions). These means were then divided into quintiles for analysis. The internal consistency (Cronbach's alpha) of the indices were in the range of 0.68 to 0.88, except the index of control over work pace (0.57).

At follow-up, the respondents were asked whether they had changed work or work tasks after they completed the first questionnaire.

### Measures of background factors

At baseline, age, gender, and a series of factors related to the private sphere, including marital status, number of preschool children (< 6 years), pregnancy, and health problems, were recorded. The question about long-term health problems was worded: 'Do you have any kind of long-term or chronic health problem (for instance, asthma, arthritis, chronic pain)? ' Optional answers were: 'no such problem', 'yes, but it does not bother me', 'yes, it bothers me somewhat', and 'yes, it bothers me a lot'.

### Statistical analyses

Statistical analyses were conducted with the Statistical Package for Social Sciences (SPSS) version 11.0. Chi-square test, Fisher's exact test, and logistic regression analysis were used to examine the relationship between work factors and the occurrence of smoking cessation. The effects of work factors were first examined in univariate analyses. Work factors that were significantly related (p < 0.05) to the occurrence of smoking cessation in these univariate analyses, were entered in a logistic regression model together with the control variables. Daily consumption of cigarettes at baseline, age, gender, marital status, having preschool children, and pregnancy were á priori chosen as control variables. As the number of pregnant respondents turned out to be very low, pregnancy was not included in the logistic regression model as originally planned. The fit of the model was assessed by Hosmer and Lemeshow test and the overall rate of correct classification.

The number of male respondents was low. As preliminary analyses showed no significant interaction between gender and work factors or between gender and other background factors with respect to effects on cessation rate, the main analyses were conducted with both genders included.

## Results

### Univariate analyses

The relationship between background factors and the occurrence of smoking cessation is presented in Table 1. Smoking cessation was most frequent in respondents who were less than 30 years of age, and least frequent in those who were 40–49 and 50–59 years of age. Married or cohabiting respondents were more likely to stop smoking than singles. Respondents with preschool children were more likely to stop smoking than respondents without preschool children. Respondents who were smoking less than 10 cigarettes per day at baseline were more likely to stop smoking than respondents who were smoking 10 cigarettes per day or more.

The relationship between working conditions and the occurrence of smoking cessation is presented in Tables 2, 3, 4 and 5. These analyses showed that the proportion of respondents who had stopped smoking at follow-up decreased with increasing hours of work per week. Work factors other than hours of work per week were not significantly associated with the occurrence of smoking cessation, but the occurrence of smoking cessation tended to be lower in respondents who had been exposed to bullying at work during the previous six months (P = 0.067). The occurrence of smoking cessation was about the same in those who changed work or work tasks between baseline and follow-up as in those who did not change work or work tasks (11.8 % vs. 9.6 %; P = 0.203).

**Table 2:** Practice area, by the proportion of respondents who had stopped smoking at follow-up. In Norwegian nurses' aides.

		Stopped smoking		
			
Practice area ^†^	N	n	Row %	P ^‡^
Somatic hospital department (adults)	241	27	11.2	0.493
Psychiatric hospital or hospital department (adults)	193	19	9.8	0.957
Paediatric department	27	2	7.4	0.656
Nursing home	966	96	9.9	0.981
Old people's home or apartment unit	232	21	9.1	0.627
Community nursing	332	31	9.3	0.684
Institution or apartment unit for mentally handicapped	252	26	10.3	0.838
Other	127	9	7.1	0.266

N = Total number if respondents in each category. n = number of cases in each category. Row % = proportion of cases in each category. ^† ^The categories are not mutually exclusive; some respondents were working in more than one workplace. ^‡ ^P-values in chi-squared test, in which respondents working in the noted practice area were compared with respondents not working in this area (all together).

**Table 3:** Work Schedule, commitment, and mastery, by the proportion of respondents who had stopped smoking at follow-up. In Norwegian nurses' aides.

		Stopped smoking		
			
Baseline work factors	N ^†^	n	Row %	P
Working hours per week				0.008
1–9	26	6	23.1	
10–18	234	32	13.7	
19–36	1655	160	9.7	
> 36	291	20	6.9	
				
Frequency of night shifts				0.724
Never	1060	102	9.6	
Sometimes	602	60	10.0	
Rather often	148	12	8.1	
Very often	404	45	11.1	
				
Commitment to the work unit ^‡^				0.721
1	408	36	8.8	
2	436	39	8.9	
3	375	43	11.5	
4	653	64	9.8	
5	349	36	10.3	
				
Perceived mastery of work ^‡^				0.541
1	456	54	11.8	
2	293	25	8.5	
3	547	55	10.1	
4	371	37	10.0	
5	558	50	9.0	

N = Total number if respondents in each category. n = number of cases in each category. Row % = proportion of cases in each category. ^† ^The total number of individuals included in these analyses were somewhat lower than the number of individuals who responded to the questionnaire, because not all respondents answered all questions. ^‡ ^Index divided into quintiles (1 is the lowest level).

**Table 4:** Physical work factors, by the proportion of respondents who had stopped smoking at follow-up. In Norwegian nurses' aides.

		Stopped smoking		
			
Baseline work characteristics	N ^†^	n	Row %	P
Moving patients in bed ^‡^				0.556
0	388	36	9.3	
1–4	912	89	9.8	
5–9	519	61	11.8	
10 or more	315	30	9.5	
				
Supporting patients between bed and chair ^‡^				0.246
0	353	33	9.3	
1–4	953	102	10.7	
5–9	525	59	11.2	
10 or more	282	20	7.1	
				
Handling heavy objects at work ^‡^				0.393
0	594	67	11.3	
1–4	1163	113	9.7	
5–9	229	17	7.4	
10 or more	111	10	9.0	
				
Work requires physical endurance				0.417
Never or very seldom	147	15	10.2	
Rather seldom	201	23	11.4	
Sometimes	555	65	11.7	
Rather often	671	60	8.9	
Very often or always	627	56	8.9	

N = Total number if respondents in each category. n = number of cases in each category. Row % = proportion of cases in each category. ^† ^The total number of individuals included in these analyses were somewhat lower than the number of individuals who responded to the questionnaire, because not all respondents answered all questions. ^‡ ^Times per shift.

**Table 5:** Psychosocial work factors, by the proportion of respondents who had stopped smoking at follow-up. In Norwegian nurses' aides.

		Stopped smoking		
			
Baseline work characteristics	N ^†^	n	Row %	P
Quantitative work demands ^‡^				0.320
1	537	54	10.1	
2	275	36	13.1	
3	529	49	9.3	
4	496	51	10.3	
5	390	32	8.2	
				
Positive challenges ^‡^				0.237
1	304	38	12.5	
2	605	62	10.2	
3	452	43	9.5	
4	371	27	7.3	
5	490	52	10.6	
				
Role conflicts ^‡^				0.276
1	355	39	11.0	
2	497	41	8.2	
3	325	41	12.6	
4	670	67	10.0	
5	373	33	8.8	
				
Control of work pace ^‡^				0.903
1	361	36	10.0	
2	603	56	9.3	
3	325	34	10.5	
4	566	61	10.8	
5	369	34	9.2	
				
Participation in important decisions ^‡^				0.459
1	340	31	9.1	
2	651	67	10.3	
3	322	34	10.6	
4	268	19	7.1	
5	640	70	10.9	
				
Fairness of immediate superior's leadership ^‡^				0.302
1	510	50	9.8	
2	197	20	10.2	
3	654	66	10.1	
4	265	17	6.4	
5	596	67	11.2	
				
Support from immediate superior ^‡^				0.992
1	402	41	10.2	
2	561	56	10.0	
3	279	27	9.7	
4	440	41	9.3	
5	543	55	10.1	
				
Social climate ^‡^				0.969
1	372	40	10.8	
2	452	44	9.7	
3	405	40	9.9	
4	399	37	9.3	
5	596	61	10.2	
				
Feedback about quality of one's work				0.258
Never or very seldom	357	44	12.3	
Rather seldom	434	48	11.1	
Sometimes	724	61	8.4	
Rather often	497	50	10.1	
Very often or always	203	17	8.4	
				
Rewards for well-done work				0.742
Not at all or very little	991	105	10.6	
Rather little	334	34	10.2	
Some	497	42	8.5	
Rather much	290	30	10.3	
Very much	93	8	8.6	
				
Exposure to threats and violence at work				0.851
Never or very seldom	1302	132	10.1	
Rather seldom	286	29	10.1	
Sometimes	391	36	9.2	
Rather often	187	21	11.2	
Very often or always	60	4	6.7	
				
Exposure to bullying at work previous 6 months				0.067
No	2119	217	10.2	
Yes	106	5	4.7	

N = Total number if respondents in each category; n = number of cases in each category. Row % = proportion of cases in each category. ^† ^The total number of individuals included in these analyses was somewhat lower than the number of individuals who responded to the questionnaire, because not all respondents answered all questions. ^‡ ^Index divided into quintiles; the lowest quintile is marked with 1, and corresponds with the lowest level of the index. Hence, in some variables (e.g. control of work pace), category "1" represents an unfortunate situation, and in other variables (role conflicts and quantitative work demands), it represents a positive situation.

### Multivariate analyses

In a logistic regression analysis (Table 6), there was a negative dose-response relationship between hours of work per week and the odds of having stopped smoking at follow-up. Belonging to the 40–49 and 50–59 age groups, being single, and smoking 10–19 cigarettes per day were associated with reduced odds of smoking cessation. In a supplementary logistic regression analysis, in which also the variable "exposure to bullying at work during the previous six months" was entered as covariate, the inverse association between hours of work per week and the odds of smoking cessation remained significant with approximately the same odds ratios as in the main model, whereas no significant association between exposure to bullying at work and smoking cessation was seen (odds ratio = 0.47; 95 % confidence interval = 0.19 – 1.18). We wondered also whether the high odds of smoking cessation in part-time workers was due to an impact of health problems, and conducted a supplementary logistic regression analysis with the variable "long-term health problems" entered as covariate together with the other variables. The results turned out the same (data not shown).

**Table 6:** The relationship between baseline characteristics and the odds of having stopped smoking at follow-up. In Norwegian nurses' aides.

Baseline characteristics	OR (95 % CI)
Working hours per week	
1–9	1.00
10–18	0.48 (0.17 – 1.33)
19–36	0.35 (0.13 – 0.91)
> 36	0.27 (0.09 – 0.78)
	
Age	
< 30	1.00
30–39	0.61 (0.35 – 1.04)
40–49	0.41 (0.24 – 0.70)
50–59	0.44 (0.24 – 0.78)
> 59	0.53 (0.20 – 1.45)
	
Gender	
Female	1.00
Male	0.82 (0.40 – 1.68)
	
Marital status	
Married or cohabiting	1.00
Single	0.61 (0.41 – 0.92)
	
Have preschool children	
No	1.00
Yes	0.93 (0.60 – 1.43)
	
Daily consumption of cigarettes	
1–9	1.00
10–19	0.51 (0.37 – 0.70)
20 or more	0.56 (0.25 – 1.23)

Results of one logistic regression analysis, with all covariates entered simultaneously.

OR = odds ratio. 95 % CI = 95 % confidence interval.

Numbers of individuals included in the analysis = 2165.

Overall rate of correct classification = 89.9 %.

Hosmer and Lemeshow test: chi-square = 5.998; p = 0.647.

## Discussion

In this 15 month prospective study of nurses' aides, who were daily smokers at baseline, there was a negative dose-response relationship between hours of work per week and the odds of having stopped smoking at follow-up. This association was also seen after adjustments for baseline consumption of cigarettes, age, gender, marital status, having preschool children, and long-term health problems.

### Methodological considerations

The study was based on a large, randomly selected, nationwide sample. The relative homogeneity of the cohort in educational attainment and occupation served to enhance the internal validity, as confounding by these factors may pose a problem in studies in which different occupational groups participate.

The response rate in the first data collection was not optimal (62 %). The number of dropouts between baseline and the follow-up was low (16 %), but there were some differences between respondents and dropouts. There was no difference between respondents and dropouts with respect to baseline consumption of cigarettes and the number of working hours per week.

We expect that people report reliably the number of hours they work per week. The questionnaire instruments that were used to measure psychological and social work factors have been found to have good construct and predictive validity as well as good internal consistency and test-retest reliability [37]. The questions used to assess the frequency of patient handling have also been found to have good validity [36].

In observational studies, such as the present one, people seem to report reliably whether they smoke or not [38]. Short-term periods of abstinence between baseline and follow-up were not measured, though, a fact that may have given an indirect emphasis on factors that relate to successful cessation attempts and long-term abstinence.

### The rate of smoking cessation

One-tenth of the respondents had stopped smoking at the 15-month follow-up. As clinical experience suggests that smokers in Norway are more inclined to make quit attempts in January than in other periods of the year, and because the observation period of the present study included two January months, it is possible that the cessation rate turned out higher than it would have done if the study had started at another time of the year. One should also take into account that the proportion of quitters may have been lower among those who did not respond to the follow-up questionnaire. In studies of the general Norwegian population during the period 1999–2003, 29 % of female smokers and 25 % of male smokers reported that they had tried to stop smoking during the previous 12 months [4]. We do not know the success rates, but it is well known that according to many studies, only about 10 % of smokers who make a quit attempt are smoke free one year later.

### Work factors and smoking cessation

Respondents who were working more than 18 hours per week were less likely to stop smoking than those who were working less than 10 hours per week. In earlier studies, hours of work per week were inversely associated with smoking cessation in 870 male members of Israeli Kibbutzes [24], whereas no association between hours of work and cessation rates was seen in 3606 Danish employees [29].

There are several possible explanations of the inverse association between hours of work per week and the odds of smoking cessation in nurses' aides. When the amount of occurrences and circumstances that require acting or responding during the week increases, people's mental capacity of handling other tasks may be reduced, and a focus on cessation efforts may be difficult to preserve over time. Long working hours may evoke emotional distress [39], a well-known inhibitor of smoking cessation. Long working hours may also elicit fatigue and a need for mental stimulation [40]. As the prevalence of smoking is high among nurses' aides, it is also possible that breaks during the workday, often spent together with smoking colleagues, could represent temptations and therefore a challenge for those who try to stop smoking. It is well documented that watching other people smoke may cause relapse in quitters.

One should keep in mind, though, that people's private financial situation may influence their work schedule as well as their need for mental stimulation. Economic worries may be a determinant of both long working hours (need more money) and low cessation rate (economic stress makes it more difficult to stop smoking), and could explain the inverse association between hours of work per week and the odds of smoking cessation that was seen in the present study. Working less than ten hours per week could, for example, be a marker of living in a marriage with a better economic situation – a situation in which it may be easier to stop smoking. It would have been an advantage if an assessment of the participants' financial situation and exposure to economic stress had been included in the study, and it is a weakness of the study that this was not done.

### Other predictors of smoking cessation

The study indicates that high consumption of cigarettes and being single are associated with lower cessation rates in nurses' aides. High consumption of cigarettes, which reflects a high level of addiction, has earlier been associated with low cessation rates in many studies [29,33]. Being single has also been associated with low cessation rates in other studies [35,41]. The social situation of singles, with more loneliness and less support, may be one explanation.

Respondents who were younger than 30, were more likely to stop smoking than older individuals. The relationship between age and the occurrence of smoking cessation in earlier studies is inconsistent. Green et al. found that cessation rates decreased with increasing age [25]. Albertsen et al. [29] found a U-shaped curve with highest cessation rates in the lowest and highest age groups. It is possible that changes in the state of pregnancy during the observation period may have contributed to the high cessation rate in the youngest age cohort of the present study.

### Confounding

The results may have been influenced by several background factors for which we were not able to control. Among the potential confounders are work factors other than the ones that were examined here (e.g. smoking by colleagues), whether or not the nurses' aides were trained and certified, personality traits (e.g. strive/achieve behaviour), age at starting smoking, and the smoking behaviour of partners.

### Implications

The great majority of Norwegian nurses' aides are employed at workplaces with occupational health service, and it is important that occupational health officers pay more attention to the smoking problem among nurses' aides. During consultations, employees who smoke should be advised to stop smoking [42], and they should be informed about – and offered therapies with documented effects on nicotine addiction, such as individual counselling, group counselling ("cessation courses"), nicotine replacement therapy, and bupropion therapy [43-46]. Nurses' aides with long working hours may be in need of more attention and help than others, and it is possible that a temporary reduction of the number of working hours per week could be a help in the cessation process. However, the majority of the nurses' aides who are working many hours per week are doing this because they need the money. Moreover, there is a lack of nursing personnel at many Norwegian health institutions, and reduced engagement by some workers would make it more difficult for others, and for the patients. Hence, making changes in the work schedule may be difficult to implement.

It is important that therapies with documented effects on nicotine addiction are available at the workplace [44]. One advantage of such workplace-based services is that employees do not have to travel to the programme or to dedicate their leisure time to it. This is particularly convenient to employees with long working hours. Smoking cessation courses tailored to nursing personnel should be available; researchers have identified a series of strategies to consider in planning such programmes [for review, see [43]].

In a systematic review of the literature, Moher et al. [44] concluded that institutional bans and restrictions on smoking at the workplace decrease tobacco consumption during the workday and reduce environmental tobacco smoke, whereas the effects of such actions on the total daily consumption were found to be less certain. Moher et al. [44] concluded also that formal studies have so far failed to document that comprehensive health promotion and protection programmes at the workplaces decrease prevalence of smoking, although there is strong theoretical rationale for such approaches.

## Conclusion

There seems to be a negative association between hours of work per week and the odds of smoking cessation in nurses' aides. It is important that health institutions offer workplace-based services with documented effects on nicotine dependence, such as smoking cessation courses, so that healthcare workers who want to stop smoking, especially those with long working hours, do not have to travel to the programme or to dedicate their leisure time to it.

## Competing interests

The author(s) declare that they have no competing interests.

## Pre-publication history

The pre-publication history for this paper can be accessed here:


